# Narrowband ultraviolet B phototherapy for dermatoses seen in elderly: a real-life experience

**DOI:** 10.1007/s10103-026-04850-3

**Published:** 2026-03-12

**Authors:** Ersoy Acer, Esra Ağaoğlu, Hi̇lal Kaya Erdoğan, Hi̇laL Çavuş, Belgi̇n Öztürk, Muzaffer Bi̇lgi̇n, Zeynep Nurhan Saraçoğlu

**Affiliations:** 1 Department of Dermatology, Eskişehir Osmangazi Üniversitesi Tıp Fakültesi Hastanesi, Eskişehir, Turkey; 2 Department of Biostatistics, Eskişehir Osmangazi Üniversitesi Tıp Fakültesi Hastanesi, Eskişehir, Turkey

**Keywords:** Narrowband ultraviolet B, Elderly, Psoriasis, Pruritus, Mycosis fungoides

## Abstract

Despite therapeutic advances such as biologics and small-molecule inhibitors, narrowband ultraviolet B (NB-UVB) phototherapy remains essential in dermatology. In recent years, the elderly population has been increasing worldwide, so NB-UVB will be more crucial in geriatric dermatology. In this study, we aimed to evaluate the efficacy and safety of NB-UVB in various dermatoses seen in elderly patients. This observational, retrospective study included patients over 65 who received NB-UVB for any dermatological disease between 2014 and 2024. We retrospectively reviewed the phototherapy and clinical follow-up forms of the patients. A total of 126 patients were included in this study. Fifty-two (41.3%) patients were male and 74 (58.7%) were female. The mean age of the patients was 71.9 ± 6.90 years. NB-UVB was administered most frequently to 30 (23.8%) of the patients with the diagnosis of generalized pruritus, 23 (18.3%) with mycosis fungoides, 20 (15.9%) with psoriasis, 15 (11.9%) with lichen planus, and 14 (11.1%) with prurigo nodularis. The mean number of sessions was 47.7 ± 21.4, and the cumulative UVB dose was 73.17 ± 49.87 J/cm^2^ in all patients. 68 (%54) patients achieved complete response, and 46 (%36.5) patients achieved partial response. Mild side effects were observed in 23 (18.3%) patients. None of them led to discontinuation of treatment. Antinuclear antibody (ANA) was positive in 29.4% of the patients. There was no statistically significant difference between ANA positivity and side effect development (*p* = 0.644). NB-UVB is an effective and safe treatment option for dermatoses such as generalized pruritus, mycosis fungoides, psoriasis, lichen planus, prurigo nodularis in the elderly population,

## Introduction

Ultraviolet phototherapy has been widely used for almost 100 years in various dermatological diseases [1]. Phototherapy is still an important treatment option in dermatology despite therapeutic advances such as biologics and small molecule inhibitors [2, 3]. It is an efficacious, safe, and cost-effective option [4]. Nowadays, narrowband ultraviolet B (NB-UVB) is the preferred phototherapy choice due to its better side effect profile. Dermatologists frequently use it to treat psoriasis, mycosis fungoides, vitiligo, atopic dermatitis, and pruritus. It has an anti-inflammatory and anti-proliferative effect on the epidermis and dermis by inducing apoptosis of T-lymphocytes and keratinocytes, regulating Langerhans cell function, reducing inflammatory cytokine production, and upregulating immunosuppressive cytokines [1, 5]. It is considered a safe therapeutic option, especially in special populations such as pregnant women, children, and elderly patients [1].

In recent years, the elderly population has been increasing worldwide. In the last 5 years, the population over 65 in Turkey has increased by approximately 21%, reaching about 9 million in 2023 [6]. The number of elderly patients in the field of dermatology is constantly growing. It is well known that the pharmacokinetics and pharmacodynamics of dermatological drugs change with aging. Moreover, many systemic comorbidities and polypharmacy may lead to drug-related iatrogenic complications in elderly patients. [7]. NB-UVB is an effective and safe treatment option, and it is most commonly used in dermatoses such as psoriasis, generalized pruritus, prurigo nodularis, mycosis fungoides, lichen planus, and eczema in elderly patients. However, limited data exist on the efficacy and safety of NB-UVB in elderly patients with chronic dermatoses [8, 9]. In this study, we aimed to evaluate the effectiveness and safety of NB-UVB phototherapy in various dermatoses seen in elderly patients.

## Materials and methods

This observational, retrospective study included patients over 65 who received NB-UVB for any dermatological disease in our clinic between 2014 and 2024. Eight hundred fifty-three files were examined in the phototherapy unit archive, and 126 patients' data were included in this study. All patients over 65 were included, and this study had no exclusion criteria.

Before phototherapy, all patients underwent a complete body examination for malignant and premalignant lesions, and a written informed consent form was obtained from each patient. We retrospectively reviewed the phototherapy and clinical follow-up forms of the patients. Sociodemographic data such as age, gender, disease duration, accompanying comorbidities and phototherapeutic data including number of NB-UVB sessions, cumulative dose of NB-UVB, antinuclear antibody (ANA) status, response to treatment and side effects were recorded. We considered more than 90% clearance of initial lesions as a complete response (CR) and clearance between 50–90% as a partial response (PR). Eskişehir Osmangazi University, Local Ethics Committee accepted the study protocol (22.10.2024, decide no:03). The principles of the Declaration of Helsinki carried out the trial.

All patients received NB-UVB with Daavlin Spectra 305/350 model UV device. Phototherapy was initiated with 70% of minimal erythema dose. The dose was augmented by 10–20% in each session. However, if minimal erythema was detected, it was continued with a 10% increase. We didn't increase the dose in the presence of persistent moderate erythema. If severe erythema and edema developed, the treatment was interrupted. After the symptoms regressed, we continued it at 50% of the last dose and increased the subsequent dose by 10%. Treatment was started 3 times a week for each patient, and as the clinical response was obtained, the dose was reduced to 2 times a week and 1 time a week. We gave maintenance treatment to patients who received a 50% response and continued the last NB-UVB dose throughout the maintenance treatment. Finally, we stopped it after giving approximately 2–4 sessions once a week. Patients were followed up for at least 6 months after treatment.

## Statistical analysis

IBM SPSS Statistics 21.0 (IBM Corp., Armonk, NY, USA) was used for the analysis. We realized descriptive analysis of all the variables. The quantitative variables were expressed as the mean ± standard deviation or median and range. The qualitative variables were expressed as an absolute value (n) and percentage. Chi-square analysis was used. For statistical significance, *P* < 0.05 was accepted as the criterion.

## Results

### Demographic, clinical, and treatment features of the patients

A total of 126 patients were included in the study. Fifty-two (41.3%) patients were male and 74 (58.7%) were female. The mean age of the patients was 71.9 ± 6.90. We initiated NB-UVB the patients with diagnosis of generalized pruritus (23.8%), mycosis fungoides (18.3%), psoriasis (15.9%), lichen planus (11.9%), prurigo nodularis (11.1%), neurodermatitis (3.2%), nummular dermatitis (2.4%), granuloma annulare (2.4%), pityriasis lichenoides chronica (2.4%), vitiligo (1.6%), pigmented purpuric dermatosis (1.6%), morphea (1.6%), pityriasis lichenoides et varialiformis acute (0.8%), atopic dermatitis (0.8%), lymphomatoid papulosis (0.8%), pityriasis rubra pilaris (0.8%) and subcorneal pustular dermatosis (0.8%), respectively. There was at least one comorbidity in 70.6% of the patients. The most common comorbidities were diabetes mellitus (27.7%), coronary artery disease (14.2%), thyroid disorders (7.1%), chronic kidney failure (5.5%), and malignancy (3.9%). Five patients had a history of malignancy, which are two breast cancer, one colon cancer, one liver cancer, and one lymphoma (Table 1).

**Table 1:** Demographic, clinical, and treatment features of all the patients

	Total (*n* = 126)
Gender	
Male	52 (41.3%)
Female	74 (58.7%)
Age, mean ± SD	71.9 ± 6.90
Fitzpatrick skin type	
1	7 (5.6%)
2	75 (59.5%)
3	44 (34.9%)
Duration of disease, mean ± SD (months)	51.5 ± 82.1
Diseases	
Generalized pruritus	30 (23.8%)
Mycosis fungoides	23 (18.3%)
Psoriasis	20 (15.9%)
Lichen planus	15 (11.9%)
Prurigo nodularis	14 (11.1%)
Neurodermatitis	4 (3.2%)
Pityriasis lichenoides chronica	3 (2.4%)
Granuloma annulare	3 (2.4%)
Nummular dermatitis	3 (2.4%)
Morphea	2 (1.6%)
Pigmented purpuric dermatosis	2 (1.6%)
Vitiligo	2 (1.6%)
Atopic dermatitis	1 (0.8%)
Lymphomatoid papulosis	1 (0.8%)
Pityriasis lichenoides et varioliformis acuta	1 (0.8%)
Subcorneal pustular dermatosis	1 (0.8%)
Pityriasis rubra pilaris	1 (0.8%)
At least one comorbidity	89 (70.6%)
Hypertension	55 (43.6%)
Diabetes mellitus	35 (27.7%)
Coronary artery disease	18 (14.2%)
Thyroid disorders	9 (7.1%)
Chronic kidney failure	7 (5.5%)
Benign prostatic hypertrophy	5 (3.9%)
Malignancy (2 breast, colon, liver, lymphoma)	5 (3.9%)
Cerebrovascular accident	3 (2.3%)
Asthma	3 (2.3%)
Cirrhosis	2 (1.5%)
Arrhythmia	2 (1.5%)
Hyperlipidemia	2 (1.5%)
Depression	1 (0.8%)
Bipolar disorder	1 (0.8%)
Ulcerative colitis	1 (0.8%)
Still’s disease	1 (0.8%)
Rheumatoid arthritis	1 (0.8%)
COPD	1 (0.8%)
Accompanied systemic treatment	8 (6.3%)
ANA	85 (100%)
Positive	25 (29.4%)
Negative	60 (70.6%)
Number of sessions, mean ± SD	47.7 ± 21.4
Cumulative dose, mean ± SD (J/cm^2^)	73.1 ± 49.8
Mild adverse effects	23 (18.3%)
Clinical response	
Complete response, n(%)	68 (54%)
Partial response, n(%)	46 (36.5%)
No response, n(%)	12 (9.5%)

*SD* standard deviation

The mean number of sessions in all patients was 47.7 ± 21.4, and the cumulative UVB dose was 73.17 ± 49.87. We combined NB-UVB with systemic treatment in 8 (6.3%) patients. Acitretin was combined in 4 of the psoriasis patients, methotrexate was combined in 2 of the psoriasis patients, and acitretin was combined in 2 of the lichen planus patients. All patients received moisturizing cream, including urea, and some received topical corticosteroids.

Of all patients, 68 (54%) had a CR, 46 (36.5%) had a PR, and 12 (9.5%) had no response (Table 1). After NB-UVB treatment, all patients were followed up for at least 6 months, and no relapse was observed. Detailed sociodemographic and phototherapeutic data of patients with generalized pruritus, mycosis fungoides, psoriasis, lichen planus, and prurigo nodularis are given in Table 2.

**Table 2:** Demographic, clinical, and treatment features according to diseases

	Generalized pruritus	Mycosis fungoides	Psoriasis	Lichen planus	Prurigo nodularis
Number of patients	30 (100%)	23 (100%)	20 (100%)	15 (100%)	14 (100%)
Gender					
Male	13 (43.3%)	9 (39.1%)	11 (55%)	5 (33.3%)	5 (35.7%)
Female	17 (56.7%)	14 (60.9%)	9 (45%)	10 (66.7%)	9 (64.3%)
Age, mean ± SD	74.1 ± 7.6	72 ± 7.2	72 ± 7.1	70.7 ± 7.6	70.6 ± 6.1
Fitzpatrick skin type					
1	2 (6.7%)	2 (8.7%)	1 (5%)	0	0
2	20 (66.7%)	14 (60.9%)	9 (45%)	12 (80%)	8 (57.1%)
3	8 (26.7%)	7 (30.4%)	10 (50%)	3 (20%)	6 (42.9%)
Duration of disease, mean ± SD (months)	32.3 ± 34.9	43 ± 63.7	133 ± 153	32.1 ± 44.6	45.1 ± 39.1
At least one comorbidity	25 (83.3%)	15 (65.2%)	15 (75%)	13 (86.7%)	11 (78.6%)
Accompanied systemic treatment	0	0	6 (30%)	2 (13.3%)	0
ANA					
Positive	6 (28.6%)	7 (41.2%)	7 (41.2%)	5 (50%)	1 (14.3%)
Negative	15 (71.4%)	10 (58.8%)	10 (58.8%)	5 (50%)	6 (85.7%)
Number of sessions, mean ± SD	41.5 ± 21.2	57 ± 24.6	49.1 ± 19	45.7 ± 14.1	41.9 ± 24.4
Cumulative dose, mean ± SD (J/cm^2^)	58.5 ± 39.1	99.2 ± 73.7	80.3 ± 41.9	67.4 ± 31.1	60.3 ± 43.1
Mild adverse effects	7 (23.3%)	5 (21.7%)	3 (15.0%)	3 (20%)	1 (7.1%)
Clinical response					
Complete response, *n*(%)	15 (50%)	13 (56.5%)	9 (45%)	12 (80%)	6 (42.9%)
Partial response, *n*(%)	12 (40%)	10 (43.5)	10 (50%)	2 (13.3%)	6 (42.9%)
No response, *n*(%)	3 (10%)	0	1 (5%)	1 (6.7%)	2 (14.3%)

*SD* standard deviation

### Adverse effects and status of ANA

ANA was measured in 85 patients before treatment, and 25 (29.4%) tested positive. Mild side effects such as erythema, itching, burning, and stinging were observed in 23 (18.3%) patients. None of them led to discontinuation of treatment. There was no statistically significant difference between ANA positivity and the development of side effects (*p* = 0.644).

## Discussion

Aging is a continuous biological process. During the aging process, the regeneration capacity of cells and tissues, chemical cleaning capacity, DNA repair capacity, mechanical protection, and immune response functions decrease. The skin structure is negatively affected like all other organs [10]. The importance of geriatric dermatoses is increasing with the rapid aging of the world population. The most common skin diseases in the geriatric population are skin infections, eczematous dermatitis, and pruritus [10, 11]. Physiological changes and associated comorbidities increase the potential side effects related to medications in elderly patients [7]. NB-UVB is considered an essential systemic treatment option for many dermatoses such as psoriasis, vitiligo, pruritus, atopic dermatitis, lichen planus, mycosis fungoides, pityriasis lichenoides chronica, and granuloma annulare in the elderly population [3, 12]. In a study of 37 elderly patients receiving phototherapy, the most common dermatoses were psoriasis, pruritus, prurigo nodularis, and eczema [9]. In our research, the most common dermatoses were generalized pruritus, mycosis fungoides, psoriasis, lichen planus, and prurigo nodularis, respectively.

Pruritus is an unpleasant sensation associated with the need to scratch [13]. Pruritus is the most common dermatological symptom in the elderly population and negatively affects the quality of life. It may have many causes, such as cutaneous and systemic diseases, various drugs, and neurological and psychiatric diseases. [14, 15]. An underlying cause sometimes cannot be determined in patients over 65; this chronic itching is defined as senile pruritus. Systemic agents such as antihistamines, pregabalin, gabapentin, and cyclosporine are used apart from emollients and antipruritic topical treatments in senile pruritus. However, NB-UVB is usually the first preferred treatment modality after topical treatments due to the associated comorbidities and multiple medications in the elderly [13]. NB-UVB is particularly effective in itching skin diseases such as psoriasis and eczema [15]. It is also an effective treatment for uremic pruritus [16]. In our study, 50% of the 30 patients with generalized pruritus had a CR, and 40% had a PR. A survey by Bulur et al. reported that NB-UVB was effective in 80% of elderly patients with pruritus [8].

In mycosis fungoides, various treatment options include topical potent steroids, nitrogen mustard, carmustine, electron-beam radiotherapy, interferon-alpha, and retinoids. Another treatment option for the skin is phototherapy [17]. A recent study found that NB-UVB and psoralen ultraviolet A (PUVA) were similarly effective in patients with early-stage mycosis fungoides, but PUVA was associated with more side effects [18]. Nowadays, NB-UVB phototherapy is widely used and provides 75–90–91.3.3% CR rates in early-stage mycosis fungoides patients [17–19]. In our study, 56.5% of the patients had a CR, and 43.5% had a PR. These response rates were lower than the literature, but in our study, patients had more advanced stages (15 were stage IA, 4 were stage IB, and 4 were stage IIA). Additionally, the mean age of our patients was higher, and the mean duration of the disease was longer. In a study, 90% of the mycosis fungoides patients with NB-UVB had CR, and 10% had PR. Fifty-three patients were stage IA, and 7 were stage IB [18]. Our study observed mild side effects in 21.7% of the patients, none requiring treatment discontinuation. The literature also shows that NB-UVB is a safe treatment option in mycosis fungoides patients [17–20].

Psoriasis is a chronic immune-mediated inflammatory skin disease that begins in two peak periods, between the ages of 16–20 and 57–60 [21]. A study determined that psoriasis started at ≥ 60 in 3.2% of patients [22]. Management of psoriasis in the elderly is a significant public health problem due to the ever-increasing elderly population and the chronic course of psoriasis [23]. In elderly patients with severe psoriasis, phototherapy, oral retinoids, methotrexate, and biologics are recommended as first-line treatments [23, 24]. Physical examination, laboratory tests, drug history, comorbidities, concomitant medicatians, caution to infections and compliance should be reviewed before selecting a treatment agent [24]. Oral retinoids are hyperlipidemic, methotrexate is myelosuppressive and hepatotoxic, and biologics are immunosuppressive agents. However, phototherapy is quite a safe treatment choice for psoriasis of the elderly [21, 23]. A study showed that NB-UVB provided complete clearance/minimal disease in 75% of patients with psoriasis and reduced the need for topical treatments [25]. There is no comprehensive data in the literature evaluating the effectiveness and safety of NB-UVB in elderly psoriasis patients. A study reported that NB-UVB provided more than 75% clearance in 73.7% of elderly patients with psoriasis [8]. In our study, 45% of patients with psoriasis achieved CR, and 50% achieved PR. Elderly patients have more comorbidities and polypharmacy. NB-UVB is relatively safe, and elderly onset psoriasis have generally mild disease so that NB-UVB may be a considerable therapeutic option in elderly psoriasis patients [22].

Lichen planus most commonly affects middle-aged adults, and generalized itchy eruption occurs in about one-third of cases. [26, 27]. In recent years, an increase in lichen planus cases has been reported in association with the COVID-19 pandemic and SARS-CoV-2 vaccination [26]. First-line treatments for cutaneous lichen planus are topical corticosteroids, intralesional corticosteroids, systemic corticosteroids, acitretin, and cyclosporine. NB-UVB is classified as a second-line treatment [27]. NB-UVB is effective in generalized lichen planus [28]. As in other dermatoses, NB-UVB is a crucial treatment option for generalized lichen planus in the elderly. There is very little data in the literature regarding the effectiveness and safety of NB-UVB in elderly generalized lichen planus. A study reported that 37.5% of elderly lichen planus patients responded to treatment [8]. In our research, response to NB-UVB was higher; 80% of patients had CR, and 13.3% had PR.

Prurigo nodularis is a chronic inflammatory disease characterized by severe itchy papulonodular eruptions (29). The disease significantly impairs quality of life and is resistant to treatment. NB-UVB is the first choice treatment modality for prurigo nodularis, which is resistant to topical treatments. It is instrumental in complex patients with systemic comorbidities and treatments [29, 30]. In our study, 43% of patients had CR, 43% had PR, and 14% were unresponsive to NB-UVB. A recent study reported that NB-UVB provided CR in 80% of patients with prurigo nodularis and PR in 20% of patients. These response rates were higher, but in this study, the mean age of the patients was 55 (18–77), and the mean disease duration was 27 months [31]. In our study, these were 70.1 and 45.6, respectively. The lower response rates may be because our patient group was older and had more chronic diseases.

NB-UVB has limitations such as availability, access, the requirement of place, staff, equipment, patient's time, and effort. However, NB-UVB is an effective treatment modality with long-term safety data, does not require laboratory monitoring, and is safe, especially for children, pregnant women, breastfeeding women, and elderly patients [3].

One of the advantages of NB-UVB is that it is suitable for combination with many treatment agents. It is practical and safe combined with topical agents such as calcipotriol, glucocorticoids, retinoids, calcineurin inhibitors, and systemic agents such as acitretin, methotrexate, biologics, and small molecules.[3, 32, 33]. In our study, We combined NB-UVB with acitretin in two psoriasis patients and methotrexate in two psoriasis patients and also combined with acitretin in two generalized lichen planus patients.

Acute side effects of NB-UVB include pruritus, erythema, tenderness, tanning, and blister formation [3]. Some authorities recommend that patients be tested for ANA before phototherapy to prevent photosensitivity and connective tissue disease exacerbation due to phototherapy. However, in recent years, it has been reported that routine ANA testing is not necessary unless there is a suspicion of autoimmune disease [34, 35]. Our study found ANA positivity in 29.4% of the patients. However, there was no difference in terms of ANA status and development of any side effects. No patient with ANA positivity developed connective tissue disease in the follow-up period. Phototherapy may increase the risk of photocarcinogenesis in the long term, and this side effect has been associated with PUVA. There is no definitive evidence that NB-UVB increases photocarcinogenesis. It is considered safe under appropriate follow-up by most experts [3].

This study had some limitations. It was a single-center retrospective design that included only the Turkish population. However, the literature has limited data on NB-UVB in the elderly population. Our results are valuable.

In conclusion, our results suggest that NB-UVB is an effective and safe treatment option for dermatoses such as generalized pruritus, mycosis fungoides, psoriasis, lichen planus, and prurigo nodularis in the elderly. With the increasing elderly population, it will become more critical. NB-UVB phototherapy may be considered a first-line treatment option in appropriate elderly patients who have resistance to topical treatments or widespread dermatoses.

## Data Availability

No datasets were generated or analysed during the current study.
